# The long-term impact of adverse caregiving environments on epigenetic modifications and telomeres

**DOI:** 10.3389/fnbeh.2015.00079

**Published:** 2015-04-08

**Authors:** Jennifer Blaze, Arun Asok, Tania L. Roth

**Affiliations:** Department of Psychological and Brain Sciences, University of DelawareNewark, DE, USA

**Keywords:** DNA methylation, miRNAs, telomeres, early-life stress, maltreatment

## Abstract

Early childhood is a sensitive period in which infant-caregiver experiences have profound effects on brain development and behavior. Clinical studies have demonstrated that infants who experience stress and adversity in the context of caregiving are at an increased risk for the development of psychiatric disorders. Animal models have helped to elucidate some molecular substrates of these risk factors, but a complete picture of the biological basis remains unknown. Studies continue to indicate that environmentally-driven epigenetic modifications may be an important mediator between adverse caregiving environments and psychopathology. Epigenetic modifications such as DNA methylation, which normally represses gene transcription, and microRNA processing, which interferes with both transcription and translation, show long-term changes throughout the brain and body following adverse caregiving. Recent evidence has also shown that telomeres (TTAGGG nucleotide repeats that cap the ends of DNA) exhibit long-term changes in the brain and in the periphery following exposure to adverse caregiving environments. Interestingly, telomeric enzymes and subtelomeric regions are subject to epigenetic modifications—a factor which may play an important role in regulating telomere length and contribute to future mental health. This review will focus on clinical and animal studies that highlight the long-term epigenetic and telomeric changes produced by adverse caregiving in early-life.

## Introduction

The time immediately after birth is a sensitive period during development that is characterized by rapid, continuing neural maturation. During this time, the brain is especially susceptible to environmental insult, and humans, non-human primates, and rodents are extremely dependent on adequate caregiving that will ensure survival and proper development (Liu et al., 1997, 2000; Caldji et al., 1998; Gunnar, 1998; Sanchez, 2006; Kaffman and Meaney, 2007; Lupien et al., 2009). Particularly, maternal care is essential for the developing organism because it provides fundamental needs such as temperature regulation, nutrition, and stimulation. Alterations to any one of these aspects of caregiving can have damaging, long-term neurobiological and behavioral impacts.

Childhood maltreatment is known to confer susceptibility to various psychiatric disorders, including depression, anxiety, and post-traumatic stress disorder (PTSD; Edwards et al., 2003; Teicher et al., 2003; Cicchetti and Toth, 2005; Anda et al., 2006). Aberrant patterns of maternal care in animals can also disrupt developmental processes and contribute to abnormal brain function and behavior (Kaffman and Meaney, 2007; Tottenham, 2014). While the initial insult takes place during early infancy, behavioral effects and psychopathology often do not emerge until later in life—that is, after a substantial delay. Whereas the precise molecular mechanisms underlying these effects are still unknown, recent work has offered considerable evidence suggesting that the path from early-life trauma to future psychopathology may be mediated by epigenetic changes. Studies continue to demonstrate that early-life stress and adversity have long-term epigenetic consequences in both humans and non-human animals.

The term epigenetics is defined as “over” or “around” genetics, referring to a broad range of factors that produce structural changes to chromatin (i.e., the DNA/histone protein complex) which can alter gene expression. Specifically, epigenetic modifications include DNA methylation and demethylation, histone modifications, and non-coding RNAs. Recent evidence has also suggested that telomeres, the end sequences that protect DNA, are also subject to the very same epigenetic modifications. This review will focus primarily on: (1) the long-term impact of DNA methylation and miRNA processing; (2) how they change in response to adverse caregiving and early-life stress; and (3) how telomeric changes offer an important avenue for future epigenetic research.

## Fundamentals of DNA Methylation

DNA methylation is an epigenetic modification that has been widely studied as a potent regulator of gene expression. This occurs through the addition of a methyl group to a cytosine (often followed by a guanine; a CG site) via the enzyme DNA methyltransferase 3a (DNMT3a; Moore et al., 2013). While DNMT3a catalyzes the reaction to create new methylation patterns, another isoform of the protein, DNMT1, mostly serves as a maintenance methyltransferase and adds methyl groups to hemi-methylated DNA. Methyl-CpG-Binding Protein 2 (MeCP2) binds to methylated cytosines and normally recruits histone deacetylases (HDACs) and other co-repressors, both of which directly and indirectly interfere with the binding of transcription factors therefore decreasing gene transcription. The addition of methyl groups and binding proteins can directly block transcription factors from binding to the DNA, but the addition of HDACs and co-repressing proteins can also promote a more tightly wound chromatin complex and less accessibility to transcription factors. In the less common case, MeCP2 can recruit CREB1 or other transcriptional activators to turn genes on Chahrour et al. (2008), although this has not been very widely studied. Though less studied, active DNA demethylation appears to be achieved by Growth arrest and DNA-damage-inducible beta (GADD45b) through a DNA-repair-like mechanism (Ma et al., 2009a,b) or by the ten-eleven translocation (TET) family of proteins, which oxidize a 5-methylctysoine to 5-hydroxymethylcytsoine (5-hmC; Li et al., 2013, 2014). In addition to hydroxymethylation, other cytosine variants are also present in DNA, including 5-formylcytosine and 5-carboxylcytosine (Brazauskas and Kriaucionis, 2014). The function of these newly-identified DNA modifications is still largely unknown, though we have recently learned that 5-hmC is responsive to changes in neural activity and learning tasks such as fear extinction training (Li et al., 2013, 2014).

Generally, methylation modifications are rapidly added or removed to DNA following environmental changes in early-life, and they are often present months or years after the initial perturbation. The mechanisms supporting the persisting or delayed effects of early-life experience on DNA methylation are still unknown, but resulting abnormalities in DNA methylation patterns in the brain and periphery have been associated with psychopathology in adulthood (for example McGowan et al., 2009; Beach et al., 2011; Perroud et al., 2013). While an important link between early-life experience, DNA methylation, and future health-related outcomes has been identified, research is continuing to examine the underlying upstream and downstream mechanisms. In the following sections, we describe studies from rodents and humans that provide evidence for this link.

## DNA Methylation in Brain

Rodent studies were the first to directly highlight the ability of caregiving experiences early in life to produce long-term and persisting changes in DNA methylation. Michael Meaney, Moshe Szyf, et al. capitalized on natural variations in rodent maternal care (licking/grooming [high-LG] and low licking/grooming [low-LG] dams (Liu et al., 1997)) to empirically test whether offspring methylation patterns were directly related to the care they received during the first week of life. They found that offspring that received low levels of maternal care in infancy (low-LG) had higher levels of methylation of the *glucocorticoid receptor* (*GR*) in the hippocampus than high-LG offspring (Weaver et al., 2004). The *GR* gene is crucial for negative feedback in the stress response (Meaney et al., 1996), and low LG offspring also showed increased corticosterone responses to a stressor. While offspring of high-LG dams showed opposite patterns of methylation and expression of the *GR* gene, effects could be reversed by cross-fostering infants. Increased *GR* methylation could also be reversed in low-LG offspring by treatment with trichostatin-A (TSA), an HDAC inhibitor. A subsequent study showed that *GR* methylation was mainly present in gene bodies, not promoters, which have different effects on transcriptional regulation (McGowan et al., 2011). Importantly, these epigenetic signatures of low levels of maternal care were present when animals were infants and persisted into adulthood, demonstrating lasting epigenetic consequences of maternal experience. Later studies even documented similar effects in the human hippocampus, including increased *GR* methylation (McGowan et al., 2009) and altered genome-wide methylation (Labonté et al., 2012) in suicide victims who experienced childhood maltreatment.

Additional studies in rodents, whereby investigators altered the gender composition within litters in order to elicit differences in maternal licking behavior have likewise shown the sensitivity of *GR* methylation to maternal behaviors (Hao et al., 2011; Kosten et al., 2014). In particular, the Kosten laboratory found that the amount of maternal licking in infancy was correlated with male and female *GR* methylation in adolescence (Kosten et al., 2014). Furthermore, this effect was present in both the hippocampus and nucleus accumbens, along with altered methylation of other genes including the *mu-opioid receptor gene* (*Oprm1*) (Hao et al., 2011).

Long-term epigenetic changes are also evident in adult rats following maternal deprivation. In this model, inadequate maternal care is elicited by simply removing the dam from the nest for a period of time during infancy. Multiple labs have used this model to show epigenetic changes at various gene loci involved in regulation of the hypothalamic-pituitary-adrenal (HPA) axis. For example, Murgatroyd et al. separated pups from the dam thirty minutes per day for the first ten days of life and measured methylation of hypothalamic *arginine vasopressin* (*AVP*; Murgatroyd et al., 2009) and pituitary *pro-opiomelanocortin* (*Pomc*; Wu et al., 2014), two genes important in regulating the release of stress hormones. Both of these genes were hypomethylated in rats that had experienced maternal deprivation, an effect that was present for at least 1 year out from the last separation. Increased *AVP* and *Pomc* gene expression were also present, as well as hypersecretion of corticosterone, passive stress coping, and memory deficits (Murgatroyd et al., 2009; Wu et al., 2014). The way in which maternal deprivation alters epigenetic marks is still being investigated, but recent studies from Spengler et al. suggest that epigenetic programming of *AVP* involves the binding of polycomb complexes (i.e., multiprotein complexes important for gene repression) and TET proteins (Murgatroyd and Spengler, 2014).

Beyond these changes, maternal deprivation also produces neurobiological alterations to other key elements of the stress response system. Maternally deprived rats show hypomethylation of hippocampal *corticotropin-releasing hormone* (*CRH*) and increased *CRH* mRNA in adulthood (Wang et al., 2014). These epigenetic changes correspond to deficits in memory and synaptic functioning that can be reversed in adulthood with environmental enrichment or blockage of CRHR1 signaling (Wang et al., 2014). Other studies have also found differences in methylation of *CRH* and its receptor in the cortex (Franklin et al., 2010) and PVN (Chen et al., 2012) after exposure to maternal deprivation in infancy. Taken together, these studies suggest that epigenetic regulation of genes governing multiple components of the HPA axis/stress responsivity is a major part of early-life stress-induced CNS epigenetic and behavioral alterations.

Our lab has characterized some of the epigenetic effects produced by early-life stress in an animal model of caregiver maltreatment. By restricting a lactating dam’s bedding material in a novel arena, we can elicit adverse pup-directed caregiving behaviors, including stepping on, dragging, dropping, and actively avoiding pups (Roth and Sullivan, 2005; Roth et al., 2009; Blaze et al., 2013). Nurturing behaviors including hovering, nursing, and licking/grooming pups are less frequent. Foundational studies in our lab demonstrated that rats who experienced early-life caregiver maltreatment had increased methylation of the *brain-derived neurotrophic factor* (*bdnf*) gene in the prefrontal cortex (PFC) as a whole (Roth et al., 2009). *Bdnf* is critical for neurodevelopment and plasticity and has been implicated in various psychiatric disorders (Martinowich et al., 2007), and maltreated-rats also showed decreased *bdnf* mRNA. Some epigenetic changes were long-lasting, appearing for one region of the *bdnf* gene (exon IX) 24 h after the last caregiver manipulation and persisting through adulthood. Moreover, maltreated-female rats showed aversive caregiving behaviors toward their own offspring, and their own offspring in turn showed similar PFC *bdnf* methylation alterations.

**Figure 1:**
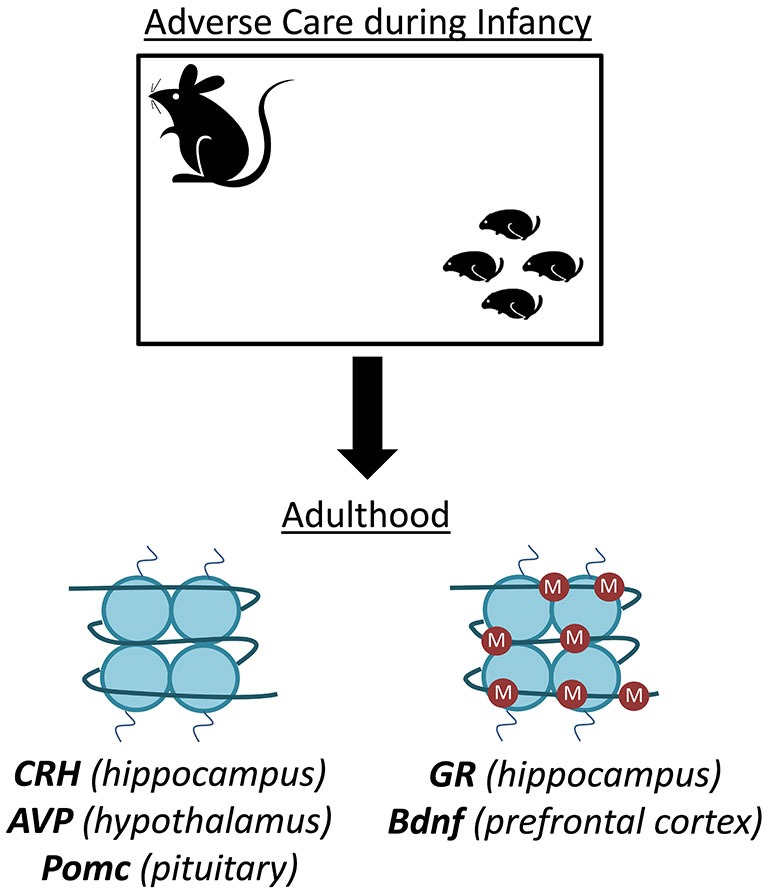
**Disruptions in infant care produce DNA methylation alterations in the rodent brain**. Both maternal deprivation and aberrant care in rodents lead to altered DNA methylation profiles at numerous gene loci important in stress, emotion, and cognition. For example, offspring that experience this stress display hypomethylation of *corticotropin releasing hormone (CRH)* in the hippocampus, *arginine vasopressin (AVP)* in the hypothalamus, and *pro-opiomelanocortin (Pomc)* in the pituitary. Conversely, the same stress produces hypermethylation of the *glucocorticoid receptor (gene)* in the hippocampus and *brain derived neurotrophic factor (Bdnf)* in the prefrontal cortex (PFC).

Later studies revealed prominent methylation alterations in the adult mPFC, dorsal and ventral hippocampus, and central/basolateral nucleus of the amygdala (Blaze et al., 2013; Roth et al., 2014). We have also demonstrated that the epigenetic effects of maltreatment (at least at the level of *bdnf* DNA methylation and gene expression) can be reversed in the adult rat PFC by zebularine treatment, an inhibitor of DNA methylation (Roth et al., 2009). Finally, we have also highlighted dynamic (i.e., alterations that are short-lived) and later-emerging (not present early in early development but later) DNA methylation alterations following exposure to caregiver maltreatment, effects that are not common in the early-life stress literature (Roth et al., 2009, 2014; Blaze and Roth, 2013; Blaze et al., 2013). For example, while *bdnf exon IV* PFC methylation was lower in adolescent maltreated-females compared to controls, it was higher in adult maltreated-females (Blaze et al., 2013). While infant and adolescent maltreated-females show higher levels of methylation associated with *bdnf IV* in the ventral hippocampus, this effect is not present in adults (Roth et al., 2014). While infant and adolescent maltreated-males do not show any changes in methylation associated with *bdnf IV* in the amygdala, adults show lower levels of methylation (Roth et al., 2014). Further, we have also observed lower levels of mRNA for key epigenetic regulatory genes in maltreated-animals when adult, an effect not detected earlier in development (Blaze and Roth, 2013). Together, our data and that of others make it clear that early-life stress in the form of disrupted caregiving has the capacity to alter central nervous system DNA methylation and gene expression (Figure 1). But, are these changes detectable in the periphery?

## DNA Methylation in the Periphery

An obstacle in translating basic DNA methylation findings to the clinic is finding parallels between the brain and the periphery, where biological measures are much easier to obtain. Peripheral DNA methylation patterns in humans are potentially promising as biomarkers for identifying psychiatric disorders (Fuchikami et al., 2011; D’Addario et al., 2012; Murphy et al., 2013; Guintivano et al., 2014). While early-life adversity is associated with altered DNA methylation in blood and buccal cells of children 7–10 years of age at time of assessment (Naumova et al., 2012) and adolescents 15 years of age at time of assessment (Essex et al., 2013), a fascinating observation is that such alterations can be present in older adults assessed several decades after the initial insult. Consistent with the rodent literature, methylation of DNA associated with genes implicated in psychiatric disorders including *GR* (Perroud et al., 2011; Tyrka et al., 2012), FK506 binding protein 5 (*FKBP5*; Klengel et al., 2013), the *serotonin transporter gene* (*5HTT*; Beach et al., 2011), and *bdnf* (Perroud et al., 2013) is altered in adults that experienced adversity (i.e., maltreatment, low socioeconomic position early in life).

While both rodent and human studies have emphasized the capacity of the *GR* gene in the brain to be epigenetically modified following early-life experiences, one study has extended these findings to the periphery. Using self-reports of early-childhood experience, Tyrka et al. found that the lack of adequate nurturing care in childhood was associated with increased methylation of *NR3C1* (human analog of the *GR* gene) promoter in blood in adults 18–59 years of age (Tyrka et al., 2012). Furthermore, increased methylation correlated to an attenuated cortisol response during a stress challenge in subjects with a history of maltreatment. Another study found a similar relationship between *NR3C1* methylation and childhood sexual abuse in leukocytes from patients (~20–40 years of age) diagnosed with major depression and bipolar disorder (Perroud et al., 2011). *FKBP5*, a gene that interacts with GRs to alter functioning of the stress response, has also been shown to be epigenetically altered with early-life stress. Klengel et al. found that adults (~30–50 years of age) carrying a risk allele for the *FKBP5* gene and that experienced childhood abuse exhibit altered *FKBP5* methylation (Klengel et al., 2013). Collectively, these findings, along with those in the rodent literature, are empirical support for parallels between the brain and the periphery.

Another gene that is widely influenced by early-life experiences and implicated in mood disorders is the *serotonin transporter gene* (*5HTT or SLC6A4*). This gene is a major player in serotonergic neurotransmission, and low levels of *5HTT* mRNA and protein have been associated with psychopathology (Kenna et al., 2012). To date, there are no long-term epigenetic studies in rodents at this gene locus, but females from the Iowa Adoptee Study have provided data on the effect of child abuse on *5HTT* methylation in the periphery (Beach et al., 2010, 2011). Adult (~40 years of age) female participants who experienced childhood sexual abuse had hypermethylation of the *5HTT* promoter in cultured cells derived from blood. Methylation levels also correlated with the severity of Antisocial Personality Disorder symptoms in abused individuals. While more studies are needed to better understand the effect of stress on *5HTT* (especially in rodents), hypermethylation of these gene may be one mechanism by which childhood maltreatment confers risk for psychopathology (at least in adult females). In a study using rhesus macaques, maternally deprived infants showed increased methylation of *5HTT* (and decreased *5HTT* mRNA) in peripheral blood mononuclear cells (PBMCs) compared to maternally reared infants (Kinnally et al., 2010). Additionally, adolescent macaques that experienced maternal deprivation are known to show altered genome-wide methylation in both the PFC and peripheral t-cells (Provençal et al., 2012), further showing parallels between the brain and the periphery.

The link between early-life stress, peripheral DNA methylation, and psychiatric disorders was further explored in a breakthrough study by Perroud et al., demonstrating epigenetic responses to behavioral therapy in borderline personality disorder (BPD) patients approximately 30–50 years of age (Perroud et al., 2013). Patients with BPD had significantly greater methylation of *bdnf* in peripheral blood leukocytes compared to controls, and the number of maltreatment episodes in childhood positively correlated with methylation levels. Intriguingly, 4 weeks of dialectical behavior therapy decreased *bdnf* methylation in patients that responded positively to treatment, while non-responders still showed higher methylation. These observations support the utility of DNA methylation as a biomarker in the clinic.

Thus far, we have discussed various candidate genes that illustrate parallels between the brain and periphery, but long-term peripheral alterations in DNA methylation across the genome have also been found after adverse early-life experiences. For example, adult males (~56–70 years of age) with depressive symptomology that were separated from their parents during childhood had greater genome-wide DNA methylation in whole blood compared to both non-stressed and healthy males (Khulan et al., 2014). Therefore, similar to the brain, genome-wide changes in DNA methylation are present in the periphery after adversity and they may be long-lived, though we point out that longitudinal studies with multiple time-points are needed to provide empirical support for this notion.

## Fundamentals of miRNAs

Another mode of epigenetic regulation is miRNAs which are short, single stranded RNAs (usually about 22 base pairs in length). Unlike DNA methylation, miRNAs contribute to gene silencing *post-transcriptionally* by binding to a target mRNA (Ambros, 2004; Bartel, 2004; Winter et al., 2009; Blaze and Roth, 2012). Primary miRNAs are produced in the nucleus and subsequently cleaved into shorter segments by a microprocessor complex containing Drosha and DiGeorge critical region 8 (DGCR8) before being transported to the cytoplasm via the Exportin-5 pathway. In the cytoplasm, the immature pre-miRNA is further cleaved by Dicer, an RNase III that is crucial for production of mature miRNAs. The mature miRNA is then loaded into an Argonaute (Ago) protein [part of the RNA-induced silencing complex (RISC)], which binds to the target mRNA to exert its gene silencing effects via degradation or destabilization of mRNA leading to translational inhibition (for a detailed figure of miRNA biogenesis, see Blaze and Roth, 2012). Certain miRNAs are restricted to a specific brain region, cell type, or developmental time point (Adlakha and Saini, 2014). Additionally, one miRNA can target multiple mRNAs and* vice versa*, suggesting that these small RNAs are part of a complex network of gene regulating elements (Adlakha and Saini, 2014).

## miRNAs in Brain

While there is considerably more knowledge about the role of miRNAs in the learning and memory and adult stress literature (reviewed in Blaze and Roth, 2012), only a few studies to date have investigated the impact (whether short- or long-term) of early-life stress on miRNAs. These studies have demonstrated that maternal deprivation in infancy increases specific miRNAs. First, Uchida et al. found higher expression of mature *miR132*, *miR124*, *miR9*, and *miR29a* in the mPFC of adult rats that experienced maternal deprivation (Uchida et al., 2010). These rats also showed a greater HPA axis response to stress and increased anxiety- and depressive-like behaviors. Behavioral and miRNA findings correlated to an increase in expression of REST4 (repressor element-1 silencing transcription factor), a protein that silences target genes. When REST4 was overexpressed in adult rats, there were similar increases in miRNAs and various target mRNAs as produced by maternal deprivation, but behavioral deficits were not present. While it is unknown which target genes were affected by the increase in individual miRNAs, REST-controlled miRNAs may be another mechanism by which stress vulnerability is translated after early-life stress (Uchida et al., 2010).

A later study likewise demonstrated stress vulnerability after maternal deprivation but instead showed the upregulation of another miRNA, *miR-504* in the nucleus accumbens (Zhang et al., 2013). Adult rats that experienced maternal deprivation and chronic unpredictable stress had higher *miR-504* levels that correlated negatively with expression of dopamine receptor D1 (*DRD1*) and D2 (*DRD2*) mRNA in the nucleus accumbens. Stressed rats with increased *miR-504* expression also exhibited depressive-like behaviors, suggesting that the gene-suppressing effect of *miR-504* on dopamine receptor expression could be mediating the effect of early-life stress on adult behavior (Zhang et al., 2013). Maternal deprivation has also been associated with long-term changes in other miRNAs. Adult rats that experience separation from the dam display higher levels of *miR-16* (correlated with lower *bdnf* expression) and *miRNA Let-7a* (correlated with lower *serotonin receptor-4* expression) in the hippocampus (Bai et al., 2012, 2014). Notably, these molecular changes correspond to increased depressive-like behaviors, suggesting that miRNAs may play a role in the development of depression associated with early-life stress (Bai et al., 2012, 2014). Together, these studies provide evidence linking miRNA regulation of gene expression to an adverse caregiving environment, and highlight this as an important area for future research.

## Fundamentals of Telomeres

**Figure 2:**
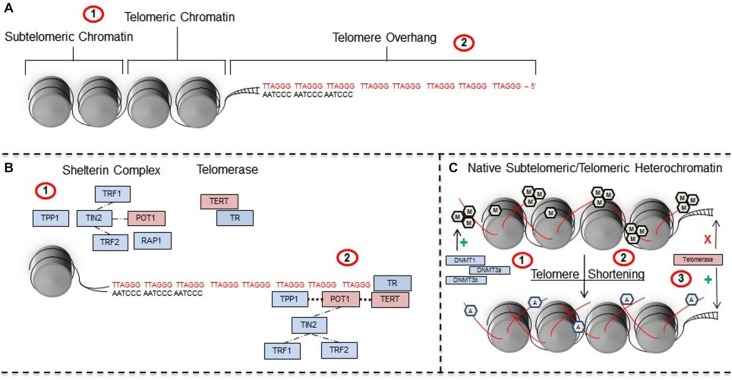
**Telomeres are regulated by telomeric-specific proteins and are subject to epigenetic regulation. (A)** Telomeres are TTAGGG nucleotide repeats that (A1) contain a subtelomeric region and telomeric region that surrounds chromatin, in addition to (A2) a 50–300 bp overhang on the 3’ strand of DNA. (A2) Stress can induce shortening of telomeres whereas telomerase promotes elongation. **(B)** Telomere length is regulated by proteins within the (B1) shelterin and telomerase complexes. Shelterin proteins have a crucial role in recruiting and positioning (B2) telomerase RNA (TR) and telomerase reverse transcriptase (TERT) on the ends of telomeres during maintenance and repair. **(C)** In their native state, (C1) telomeres are in a hypermethylated state that is regulated and maintained by key DNA methyltransferases including DNMT1, DNA methyltransferase 3a (DNMT3a), and DNMT3b. However, (C2) telomere shortening induces a shift to a euchromatic state involving increased acetylation and decreased methylation which (C3) facilitates the recruitment of telomerase to telomere ends. Abbreviations: TTAGGG-repeat binding factor 1 (TRF1), TTAGGG-repeat binding factor 2 (TRF2), collective acronym of previous labels TINT1, PTOP, and PIP1 (TPP1), TRF-1 interacting nuclear protein/factor 2 (TIN2), (RAP1), protection of telomeres 1 (POT1), telomerase reverse transcriptase (TERT), TR, methyl group (pentagonal M), acetyl group (pentagonal A).

Another biomarker for measuring the impact of early-life stress is telomere length, which is also subject to epigenetic modifications. Telomeres are long stretches of TTAGGG nucleotide repeats located at the ends of chromosomes that function to protect genomic DNA from damage following replication. They are primarily double stranded except near the terminal ends where there is a 50–300 base pair overhang of the 3’ TTAGGG strand (Figure 2A). With each cell-division, telomeres naturally shorten in length (i.e., the “end replication problem” (Blackburn, 1991) before reaching a critical point which triggers replicative senescence. Given that telomeres continually shorten with each cell division throughout the lifespan, the length of telomeres has been proposed as a measure of disease-risk (e.g., cardiovascular disease) and biological aging. However, studies investigating its utility as a measure for biological aging have been discrepant (von Zglinicki and Martin-Ruiz, 2005; Mather et al., 2011; Fossel, 2012).

Although the base-pair sequence of telomeres (i.e., TTAGGG) is conserved across mammalian species, telomere length can substantially vary across different cell-types and between mammals, including those commonly employed for investigating the impact of early-life stress (i.e., humans (~8–20 kilobases (kb)), mouse strains (~30–150 kb), and rat strains (~20–100 kb) (Cherif et al., 2003; Samassekou et al., 2010). In humans, telomeres shorten quickly (~170–270 bp/yr.) after birth (0–3 years of age), before eventually plateauing to levels observed in adults (~30–50 bp/yr.) (Slagboom et al., 1994; Zeichner et al., 1999). Whereas previous studies have not detected differences in telomere length (in white blood cells, umbilical artery, and skin) between male and female human newborns (Okuda et al., 2002) recent work has identified differences in telomere length of newborns (in bloodspots) across race and sex (Barrett and Richardson, 2011; Drury et al., 2015). Consistent with these recent findings, other studies have also identified differences in telomere length between race and sex during adolescence (Zhu et al., 2011) and following early life-stress (Drury et al., 2012, 2014; Price et al., 2013).

## Telomere Length in the Periphery

Research investigating telomere changes following psychological stress is relatively new (Epel et al., 2004) and its use as a biomarker for measuring the biological impact of early-life adversity is even more recent (for a review see Shalev, 2012; Price et al., 2013). Retrospective reports examining instances of child maltreatment, abuse, or neglect have demonstrated that early-life stress is associated with the accelerated shortening of telomeres in peripheral cells (i.e., peripheral blood mononuclear cells and buccal cells) that is present at both an early age and adulthood. Specifically, Tyrka et al. (2010) and Kiecolt-Glaser et al. (2011), found that 26.9 (*SD* = 10.1) and 69.7 (*SD* = 10.14) year old adults, respectively, who reported (via the childhood trauma questionnaire) physical and emotional neglect during childhood exhibited shorter telomeres in peripheral blood mononuclear cells (Tyrka et al., 2010). Kananen et al. (2010) expanded on this to show that the number of adverse events (e.g., financial difficulties, substance abuse problems, mental-health issues, conflicts, etc.) during childhood was strongly associated with shorter telomeres in peripheral blood from older adults, 48–87 years of age, with anxiety (Kananen et al., 2010). Chen et al. (2014) reported similar findings by illustrating how the number of adverse childhood events experienced before the age of 18 was correlated with shorter leukocyte telomeres in adults (Chen et al., 2014). Together, the work of Kananen et al. (2010) and Chen et al. (2014) highlight a possible dose-response relationship between increased adversity and reduced telomere length in adulthood, but more research is needed to better understand this relationship. Interestingly, O’Donovan et al. also found an association between childhood trauma and shorter telomeres in leukocytes, but only in individuals with PTSD at 30.5 (*SD* = 10.14) years of age—suggesting that telomeres shortening following early-adversity may be associated with mental-health related disorders in adulthood (Kananen et al., 2010; Tyrka et al., 2010; Kiecolt-Glaser et al., 2011; O’Donovan et al., 2011; Shalev et al., 2014). Overall, these studies show that adverse experiences during childhood are associated with shorter telomeres in adulthood, but they also exemplify that telomere shortening is apparent many decades after the initial insult.

While the impact of early-life stress on telomeres is evident during adulthood, accumulating evidence suggests that these changes may manifest at a much earlier age. Drury et al. (2012) found that the length of time severely neglected children spent under institutional care (i.e., Romanian orphanages) was associated with shorter buccal cell telomeres between 1.8 and 4.5 years of age. In collaboration with Mary Dozier, our lab has since found that children at a heightened risk for maltreatment (identified by child protective services) exhibit shorter telomeres in buccal cells than low-risk children by age 5 (Asok et al., 2013). Furthermore, Shalev et al. (2013) determined that exposure to physical violence (i.e., violence between the mother and partner, bullying, or physical maltreatment) predicted faster telomere attrition in buccal cells—where exposure to two or more acts of violence significantly accelerated this attrition by 10 years of age. Drury et al. (2014) recently found that children between 5–15 years of age who experienced adverse familial experiences (i.e., witnessing violence, dealing with parental incarceration, or suicide) also exhibited shorter salivary telomere length than children who did not experience any adverse experiences. Considered together with retrospective reports obtained during adulthood, these studies highlight: (1) a consistent relationship between various forms of childhood adversity and shorter telomeres; (2) evidence that telomere shortening may occur rapidly and could be present across development; and (3) that telomere shortening may be related to future mental-health related changes.

Given that environmental factors play a crucial role during child development (Cicchetti and Toth, 2005; Toth et al., 2013), it is important to note that some studies have investigated the role of caregiving on telomere length (Theall et al., 2013). Drury et al.’s work has highlighted the importance of proximal environmental factors such as family. Importantly, they have also found differences on the impact of early trauma across gender, showing girls may be more susceptible (Drury et al., 2012, 2014). Recent work has also shown that lower paternal care in males (23.4 years of age *SD* = 1.6) and lower maternal care in females (23.5 years of age *SD* = 1.9) predicted shorter leukocyte telomere length in adults (Enokido et al., 2014). Additionally, reported non-supportive parenting in African-American youth (21.8 years of age *SD* = 1.15) was associated with shorter leukocyte telomere length (Beach et al., 2014). Consistent with these studies, we have found that increased parental care, in the form of increased parental responsivity/sensitivity, appears to buffer high-risk children from accelerated telomere shortening (Asok et al., 2013). Thus, it is possible that interventions targeting primary caregiving in early-life may have a protective role on the long-term detriment to telomeres following early-adversity (Chen et al., 2014).

## Telomere Length in the Brain

Human studies have been important for examining the environmental factors (i.e., caregiving) that may moderate the long-term impact of early-life stress on telomere length in the periphery. Research using rodent models has extended our understanding of whether early-life stress induces long-term telomeric changes in the brain. Botha et al. (2012) found that male rats who experienced maternal deprivation (i.e., removal of the mother for 3 h/day) from postnatal days 2–14 had longer telomeres in cells of the ventral hippocampus at postnatal day 65 (Botha et al., 2012). We have recently shown that caregiver maltreatment (i.e., mothers showing more stepping-on, dragging, dropping, and reduced nursing behaviors) produces longer telomeres in cells of the amygdala in maltreated-female rats at postnatal day 90 (Asok et al., 2014). Interestingly, we did not observe differences in maltreated-male rats within the amygdala, PFC, or ventral hippocampus. Conversely, females that received nurturing care showed longer telomeres in the PFC relative to maltreated-females. These data are an important extension to human studies in that they parallel the long-term effects of adversity on telomere length and illustrate differences on the impact of trauma across sexes (i.e., a pronounced impact on females). Furthermore, these studies also point to a more interesting phenomena—that telomere length in the brain may be inversely related to the shortening observed in the periphery following early-life stress (Thomas et al., 2008). Whereas alterations in cellular proliferation have been suggested to account for telomere changes in the brain, it is unknown in which type of cells (i.e., neurons or glia) these changes are occurring (Dabouras et al., 2004; Thomas et al., 2008). More research is needed to uncover the relationship between peripheral and central changes to telomere length following early-life stress and in psychopathology (Eitan et al., 2014).

### Telomere Maintenance and Regulation

While it is clear that early-life stress is associated with changes in telomere length in the peripheral and central nervous systems, the mechanisms regulating these changes are still unknown. Recent work has shown a positive correlation between the number of adverse child experiences and increased telomerase activity—a proposed compensatory mechanism for increased damage to telomeres (Chen et al., 2014). Telomere length is primarily maintained by the enzyme telomerase, but also by a protein complex known as shelterin. Telomerase consists of two main proteins: the catalytic protein telomerase reverse transcriptase (TERT) and a primary RNA component known as telomerase RNA (TR; Figure 2B; de Lange et al., 2006). In general, TERT is responsible for the *de novo* addition of TTAGGG nucleotides a single base at a time onto the 3’ telomeric overhang (de Lange et al., 2006). The RNA TR component functions as a template for TERT activity when DNA base pairs are added during telomere elongation. The proper functioning of telomerase is dependent on the six proteins contained within the shelterin complex: TTAGGG-repeat binding factor 1 and 2 (TRF1 and TRF2), TRF-1 interacting nuclear protein/factor 2 (TIN2), repressor activator protein 1 (RAP1), TPP1 (collective acronym of the previous labels TINT1, PTOP, and PIP1), and protection of telomeres 1 (POT1; Figure 2B).

In humans, TRF1 regulates telomeres to prevent elongation. However, in mice, TRF1 is functionally necessary for elongation, and deletion causes early embryonic death (Karlseder et al., 2003). TRF2 is also an essential factor in telomere maintenance and, like TRF1, deletion leads to embryonic death (Celli and de Lange, 2005) and cellular senescence. TIN2 functions as a core component holding TRF1, TRF2, and POT1 together. Additionally, TIN2 can regulate the function of TRF1 and TRF2 in that it helps to stabilize their positioning on telomeres (Kim et al., 2004; Ye et al., 2004). RAP1 functions to control telomere lengthening, but can also regulate aspects of gene transcription and silencing (Martinez et al., 2010). TPP1 is required for telomere elongation and functions to recruit POT1 to telomeres. Finally, POT1 is thought to protect the far end of the TTAGGG strand and overall chromosome in certain circumstances (Lei et al., 2004), in addition to serving a critical role in controlling telomere length and recruiting telomerase (McCord and Broccoli, 2008). While the primary role of telomerase and shelterin is the maintenance of telomere length, other factors (e.g., Poly-ADP ribosylases, helicases, etc.) can also regulate telomeres, but their function is beyond the scope of this review (for a more detailed discussion of these factors see de Lange et al., 2006). Fundamentally, telomere elongation and attrition is differentially controlled by a delicate interaction between specific proteins contained within the telomerase and shelterin complexes. To date, only a few studies have investigated the impact of stress, in general, on telomerase (Beery et al., 2012; Zalli et al., 2014) and limited progress has been made in understanding how changes in telomerase may alter future mental-health related outcomes (Zhou et al., 2011). Furthermore, no research has investigated changes to shelterin proteins following early-life stress. Thus, future studies investigating alterations to telomerase and shelterin proteins following early-life stress are important for identifying the mechanisms that may regulate changes in telomere length.

### Epigenetic Control of Telomere Length

Interestingly, telomeres are subject to epigenetic regulation (for a comprehensive review see Blasco, 2007; Galati et al., 2013), but the relationship between early-life stress and epigenetic changes to telomeres has yet to be explored. Subtelomeric chromatin regions do not contain many genes (i.e., a few zinc-finger and olfactory receptor genes) (Riethman et al., 2004), but can still be influenced by both acetylation and methylation (Blasco, 2007). In their native state, telomeres exhibit features of heterochromatin (i.e., hypermethylation of histone 3 (H3) lysine 9 (K9) and H4K20 and hypoacetylation of H3 and H4). This hypermethylated state is regulated and sustained by DNA methyltransferases including: DNMT1, DNMT3a, and DNMT3b (Gonzalo et al., 2006) and histone methyltransferases including suppressor of variegation (SUV) 39h1 and SUV39h2 (Garcia-Cao et al., 2004). However, because telomeres are composed of TTAGGG repeats and lack CG sites they are generally not methylated.

Following telomere shortening (Figure 2C), subtelomeric and telomeric chromatin exhibit a conformational shift to a more euchromatic state with concomitant decreases in methylation and increases in acetylation (Benetti et al., 2007; Blasco, 2007). This shift in the chromatin of subtelomeric and telomeric structure is thought to facilitate the recruitment of telomerase to telomeres (Blasco, 2007). Taken together, the epigenetic control of telomeres and recruitment of telomerase may, speculatively, have an important role during specific times following early-life stress. Yet, research is needed to: (1) first understand if epigenetic changes to telomeric regions occur following early-life stress; and (2) how they may be related to future behavior (Buxton et al., 2014).

### microRNA Regulation of Telomeres

microRNAs also offer a potential target for investigating telomere control (Chen et al., 2010; Santambrogio et al., 2014), although their function in regard to telomeric changes following early life-stress also remains unclear. Research has shown that particular microRNAs target telomere associated genes; miR-138 can impact TERT levels (Mitomo et al., 2008; Chen et al., 2010), miR155 can regulate TRF1 expression (Dinami et al., 2014), and miR-186 can target TRF2 (Chilton et al., 2014). An important caveat is that many of these findings are derived from research utilizing various cell types. Chilton et al. (2014) found that specific microRNAs (miR186, miR-15a, and miR-96) and TERT mRNA were upregulated within T-cells shortly after (~60 min) acute exercise. Interestingly miR-186 and miR-96 (can target TRF2 (which serves a critical role in telomere maintenance) suggesting that acute experiences can indeed impact miRNAs that regulate telomeric factors. Whereas other mechanisms of telomere and telomere-associated gene control exist (e.g., the telomere positioning effect (TPE) and alternative lengthening of telomeres (ALT); Baur et al., 2001; Cesare and Reddel, 2010), no research has investigated telomere-related microRNA changes in response to early-life stress. Thus, microRNA regulation of telomeres offers another crucial direction for future research examining how early-life stress can lead to both short- and long-term changes in telomere length.

### Conclusions and Future Directions

A wealth of research over the last several decades has significantly enhanced our knowledge of the biological impact of early-life stress. Despite this progress, the molecular mechanisms by which caregiver experiences affect future behavior and mental health are still in the early stages of investigation. Data continue to suggest that epigenetic changes, such as DNA methylation and microRNA processing, are mechanisms by which early-life stress produces changes in the brain and periphery that can ultimately influence behavior. Evidence in recent years has also pointed to alterations in telomere length as a novel target by which the impact of early-life stress can be measured. Interestingly, telomeres and telomeric enzymes are also influenced by epigenetic changes—highlighting a rich avenue for future research. While many of these epigenetic and telomeric alterations are discussed in the literature as “long-lasting” (i.e., meaning alterations are present at multiple time-points from infancy to adulthood), without a longitudinal study design a better term may be “long-term”, as it is possible the alterations were not present early in development but emerged much later in life (as we have seen with some of our rodent work). Several groups however have documented such alterations present in infants and children exposed to adversity (for example Oberlander et al., 2008; Naumova et al., 2012; Asok et al., 2013), and future work in which data can be collected from the same subjects repeatedly over the course of time will help us understand even more about the epigenetic and telomeric consequences of adverse caregiving environments. Finally, *how* these alterations manifest in pathways of psychopathology is a research area that certainly merits further exploration.

## Conflict of Interest Statement

The authors declare that the research was conducted in the absence of any commercial or financial relationships that could be construed as a potential conflict of interest.
